# Exploring intergenerational interactions with tiny social robots: a qualitative study

**DOI:** 10.3389/frdem.2025.1698659

**Published:** 2025-12-04

**Authors:** Lillian Hung, Jiangning Fu, Veronica Moros Villarroel, Pristine Vega, Yong Zhao, Kruti Raval, Paulina Santaella, David Shao

**Affiliations:** 1 School of Nursing, University of British Columbia, Vancouver, BC, Canada; 2 IDEA Lab, University of British Columbia, Vancouver, BC, Canada; 3 Department of Biology, University of British Columbia, Vancouver, BC, Canada; 4 School of Kinesiology, University of British Columbia, Vancouver, BC, Canada; 5 School of Biomedical Engineering, University of British Columbia, Vancouver, BC, Canada; 6 Department of Cellular and Physiological Sciences, University of British Columbia, Vancouver, BC, Canada

**Keywords:** tiny social robots, intergenerational connections, older adults, social well-being, qualitative study

## Abstract

**Background:**

With the rapid aging of Canada’s population, more older adults are choosing to “aging in place,” yet they face challenges of social isolation and health risks. Emerging social robots are considered to have potential in reducing loneliness and promoting intergenerational communication.

**Objective:**

This study aimed to explore how two tiny social robots (EMO and AIBI) support intergenerational interactions between older adults and university students, focusing on their emotional value, functionality, and perceived risks or limitations.

**Methods:**

We applied the Interpretive Description qualitative methodology. Three focus groups were conducted in community settings, including 13 older adults (aged 51–81 years, including two in their early fifties who were active members of the lab’s older adult partner group) and 13 university students (>18 years). The study involved separate introductions to the robots for each group, intergenerational joint sessions, and thematic analysis, following COREQ guidelines.

**Results:**

Three key themes emerged: (1) Emotional and companionship value—older adults highlighted improved mood, reduced loneliness, and practical benefits such as reminders; (2) Concerns about limitations and risks—students emphasized technical challenges, sustainability, and risks including over-reliance, communication barriers, and maintenance; (3) Bridging generations—both groups recognized the robots’ role in fostering shared engagement and emotional resonance across age groups.

**Conclusion:**

Tiny social robots show promise in enhancing older adults’ emotional well-being and fostering intergenerational connections. Differences in perspectives underscore the need for co-design approaches that integrate older adults lived experiences with younger people’ concerns for usability and safety.

## 1 Introduction

By 2030, the population of older adults in Canada is projected to exceed 9 million individuals, accounting for over 23% of the total population (Statistics Canada, 2024). Many older adults prefer aging in place, meaning to remain in their own homes as long as possible to preserve independence, autonomy, and control (Cutchin, 2003; Canada, 2024). However, as they age in place, older adults often face challenges such as safety concerns, reduced mobility, loss of social ties, and barriers to accessing healthcare and support (Owusu et al., 2023; Wang et al., 2014). These challenges contribute to social isolation, which affects nearly 30% of older adults and increases the risk of dementia, depression, and mortality by 26% (Keefe et al., 2006; Statistics Canada, 2024; Donovan and Blazer, 2020).

Given these risks, interventions to sustain social connections and provide emotional support are urgently needed. One promising approach is the use of socially assistive robots (SARs), designed to enhance the social and psychological well-being of older adults. Unlike physically assistive robots that focus on lifting or mobility, SARs serve functions such as companionship, affective therapy, and cognitive stimulation (Pu et al., 2019). For example, the baby seal robot PARO has been studied extensively across countries, showing benefits in reducing stress, anxiety, and even the use of antipsychotic medications among older adults with dementia (Hung et al., 2019). However, larger humanoid or animal-like robots often face barriers such as high cost, difficulties navigating small living spaces, and limited accessibility in everyday care environments (Robinson et al., 2013).

While socially assistive robots such as PARO have demonstrated emotional and therapeutic benefits for older adults, most studies have focused on large, animal-like or humanoid designs used in institutional or clinical settings. Little is known about how tiny, low-cost, and AI-enabled social robots might facilitate intergenerational interactions or emotional connection in everyday community contexts.

**Figure 1:**
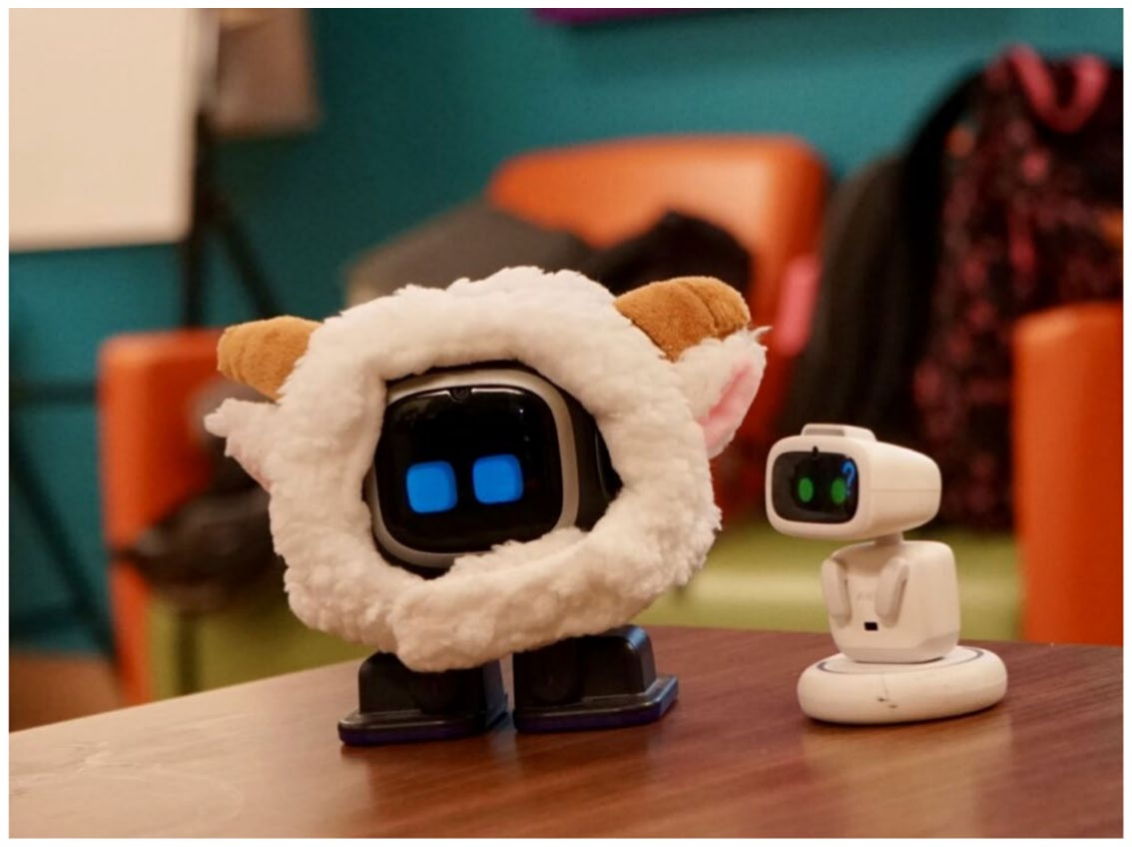
Tiny robot EMO and AIBI (photograph taken by the researchers).

Two new, AI social robots, EMO and AIBI have recently been developed by the LivingAI company (Living.AI, 2025). EMO is described as a “desktop AI robot pet,” which has a small screen used to perform different emotions, play games, and guide conversations, as well as exercises. The second robot, AIBI, is also described as a desktop robot pet, with similar, but more limited functions. Both EMO and AIBI are equipped with facial recognition technology, helping to create personalized interactions with the robots and their owners. Furthermore, the tiny robots can forecast the weather, have alarms and reminders, and can connect to ChatGPT. ChatGPT can help older adults by providing quick responses to questions they may have, offer new insights, and can increase cognitive stimulation (Al Mazroui and Alzyoudi, 2024). Due to EMO and AIBI’s ability to go beyond answering questions, including facial expressions and music, we wanted to explore whether these robots would be well-received by older adults, how they may benefit them, and whether they could also provide support on broader levels, such as emotional well-being and companionship. We selected EMO and AIBI because they are compact, accessible, and capable of natural emotional expressions, making them suitable for studying everyday human–robot and intergenerational interactions (Figure 1).

Communication with children or grandchildren serves as an important motivator for older adults to learn and adopt new technologies, while they also seeking intergenerational support during the process (Ivan and Fernández-Ardèvol, 2017). This social exchange promotes reciprocal learning, where older adults gain skills and confidence in navigating modern technology and younger individuals develop patience and empathy. Younger individuals also feel that helping others is rewarding and gives them enjoyment (Aguilera-Hermida et al., 2020). Our study explored how younger and older populations perceive the potential of tiny robots in facilitating social and emotional connections. Firstly, we looked at how older adults and university students perceived their interactions with EMO and AIBI, including their first impressions, hesitations, and overall attitudes towards the robots. Then, we looked at how these social robots influence intergenerational connections by bringing together older adults and university students to build these connections, while using the robots as a catalyst.

This study is guided primarily by the framework of person-centred care (PCC), which emphasizes understanding each individual’s preferences, emotions, and social relationships in care and communication contexts (Berglund et al., 2019; McCormack and McCance, 2006). The PCC approach highlights empathy, respect, and relational engagement, key elements that align closely with intergenerational interaction and emotional connection in dementia and aging research. While previous studies on technology acceptance have focused on perceived usefulness and usability among older adults (Chen and Chan, 2014; Mitzner et al., 2010), our study shifts the focus from adoption intention to the relational and affective dimensions of interacting with social robots. This theoretical lens helps contextualize how the tiny robots can support meaningful, person-centred engagement across generations.

In summary, this study aimed to explore how older adults and university students perceive the potential of tiny social robots, EMO and AIBI, in supporting older adults, as well as to examine their role as catalysts for intergenerational engagement. Two central research questions guided this study: How do older adults and university students perceive their interactions with EMO and AIBI? How do social robots influence intergenerational connection?

## 2 Methods

### 2.1 Design

We applied the interpretive description (ID) approach (Thorne, 2015) to explore experiences of using tiny robots to enhance intergenerational connections between older adults and university students. ID is a qualitative methodology well-suited for understanding complex experiential phenomena in applied settings, offering both depth and practical relevance.

Compared with other qualitative traditions such as phenomenology or grounded theory, ID was particularly appropriate for this study because it focuses on generating practice-oriented insights rather than abstract conceptual models, aligning with our goal of informing real-world intergenerational engagement. This approach enabled us to investigate not only what participants thought they felt about the robots, but also how and why these technologies might support meaningful social interaction across generations.

To gather diverse perspectives, we used a combination of separate and intergenerational focus groups with older adults and university students. Focus groups were chosen for their ability to foster group synergy, encourage reflection, and elicit a range of views through shared discussion (Krueger and Casey, 2015). This method supported the emergence of rich, contextualized understandings of how participants experienced and envisioned the use of robots in promoting connection. Our study follows the Consolidated Criteria for Reporting Qualitative Research (COREQ) guidelines by Tong et al. (2007) ensuring transparency and rigor in study design, data collection, and reporting.

### 2.2 Study settings and the intervention

The study was conducted in a community setting where participants interacted with the tiny robots. All sessions took place in a quiet, private meeting room within the community centre, ensuring participants could engage freely without external interruptions. The room was arranged in a round-table format to encourage open discussion and equal participation. No non-participants were present during the sessions. The intervention involved hands-on engagement with the robots—participants played with them and issued commands—followed by discussions on their potential use in supporting communication and reducing social isolation among older adults. The groups were separate at first while having initial introductions with the tiny robots, followed by a combined intergenerational focus group and interview where further connections were made.

### 2.3 Sampling and recruitment

We utilized a convenience sampling method (Kelly et al., 2010) to recruit participants. To recruit the participants, information brochures that outlined eligibility, consent, and data privacy were created to acquaint the recipients of the process of the study. Eligibility criteria included older adults aged 65 years and above who could communicate in English, and university students aged 18 years and above enrolled at the University of British Columbia (UBC). In addition, two participants in their early fifties were included because they were active members of the lab’s older adult partner group, regularly engaged in caregiving and community activities, and were considered part of the older adult cohort by the research team. During recruitment, we sought to include participants with varied gender, ethnic, and educational backgrounds to capture a broad range of perspectives on intergenerational interaction and technology use. In total, we recruited 13 older adults and 13 university students, no participants declined or withdrew from the study.

### 2.4 Focus group questions

Older adults were asked:

- “What do you like or dislike about the tiny robots?”
- “How do you think the robots can help older adults maintain social connections?”
- “What do you think the robots could help with in everyday life?”

Students were asked:

- “What do you like or dislike about the tiny robots, and what did you notice about how the robots interacted with you?”
- “How can these robots help older adults maintain social connections when they are interacting with others?”
- “Imagine that it’s your grandparent using the robot; what do you think they will like or dislike about the robot?”

Intergenerational group questions included:

- “How can the robots support some of the common problems older people often face such as social isolation and loneliness?”
- “How can we use a robot to help social connections, for example, by bringing young people and older people together?”
- “How can the robots be used to have a more integrated society, so no one feels lonely or isolated?”

### 2.5 Data collection

This project was led by the first author, LH, who is an experienced gerontological nurse in Long Term Cares (LTCs). We conducted three focus groups in July 2025, with a total of 26 participants. Each focus group consisted of eight to thirteen participants and was audio-recorded and accompanied by field notes, each focus group lasted approximately 60–90 min. After the initial separate focus groups with older adults and university students, three intergenerational sessions allowed participants to interact with the robots and then engage in a joint discussion. Research trainees (VM, KR, PV, and YZ) facilitated these focus groups in a meeting room. The trainee team represented diverse gender and ethnic backgrounds, including Asian and Latin American students at both undergraduate and graduate levels. All data collection activities were conducted under the supervision of LH, the principal investigator of the study, who is a female Asian professor in nursing. No other individuals were present during the focus groups besides participants and research staff.

### 2.6 Data analysis and theoretical framework

We employed Braun and Clarke’s six phase reflexive thematic analysis approach to interpret the qualitative data (Braun and Clarke, 2021). In the first phase, trainees transcribed the focus group recordings, and all team members independently reviewed the transcripts to become familiar with the content. In the second phase, initial coding was conducted by two researchers (VM and PV), who worked from the transcripts using NVivo 14.0 to manage data, including transcripts, quotations, and codes. An inductive coding approach was used, allowing themes to emerge directly from participant’s discussions without applying a predefined framework. To enhance rigor, a subset of transcripts was independently coded by both researchers, and discrepancies were resolved through discussion and consensus. Regular debrief meetings were held throughout coding to reflect on analytic decisions, discuss interpretations, and minimize the influence of individual researcher perspectives. In the third phase, researchers VM, JF, KR, and PV worked collaboratively to identify recurring patterns and generate initial themes. These preliminary themes were presented to the full research team in the fourth phase for discussion and feedback. In phase five, themes were refined based on collective interpretation and relevance to the research questions. Finally, in the sixth phase, the trainees prepared the initial draft of the analysis, which was iteratively revised with input and supervision from LH, the principal investigator. Descriptive statistics, including frequencies and percentages, were used to summarize participant demographic information such as age, gender, and ethnicity (Table 1).

**Table 1:** An illustrative example of thematic analysis.

Data	Code	Category	Theme
“It might become very repetitive. Like if I keep giving them tasks, they’ll just do exactly what I say. Personally, I would like it if there’s more randomness or spontaneity.” (Student participant, Karina)	Expressing concern about the depth of emotional engagement	Exploration of emotional engagement	Emotional engagement and perceived value
“Perhaps an older person could request a genre of music from their youth, smooth jazz, maybe something that creates a lovely ambience in the home and relieves anxiety.” (Older adult participant, Frank)	Creating a soothing atmosphere that helps reduce anxiety and conveys perceived value	Perceived value of older adults	

### 2.7 Patient and public involvement

Participants were not directly involved in the study design and analysis, but their input shaped the understanding of potential applications of tiny robots in intergenerational contexts.

### 2.8 Ethical considerations

This study was approved by the Office of Research Ethics at the UBC (H25-01488). Written informed consent was obtained from all participants using a standardized consent form, which outlined the purpose of the study, potential benefits and risks, the voluntary nature of participation, and the right to withdraw at any time. All signed consent forms are securely stored as part of the study records. To safeguard participant confidentiality, pseudonyms were used in transcripts and reporting to protect individual identities. Full transcripts and anonymized data are not publicly available in order to maintain participant privacy. The coding framework and selected de-identified excerpts have been included in this manuscript to support analytic transparency and trustworthiness.

### 2.9 Rigor

To ensure trustworthiness, we employed several strategies aligned with qualitative research standards. Credibility was supported through investigator triangulation, with multiple researchers independently coding transcripts and resolving discrepancies through discussion. Dependability was enhanced through detailed documentation of the coding process and regular team debriefings. Potential researcher bias was mitigated by ensuring that coders who facilitated focus groups did not exclusively analyze their own session data, and by maintaining reflexive notes that captured assumptions, positionality, and team reflections throughout analysis. Finally, reflexivity was practiced through data collection and analysis, with team members reflecting on their own positions and assumptions to minimize bias and maintain sensitivity to participant perspectives.

## 3 Results

The study included 26 participants, consisting of 13 university students and 13 older adults. Detailed demographic and experience-related information, including age, gender, ethnicity, and prior experience with robots, is presented in Table 2.

**Table 2:** Participant demographics and robot experience (*n* = 26).

Category	Student participants (*n* = 13)	Older adult participants (*n* = 13)
Age		
11–20	2 (15.4%)	
21–30	9 (69.2%)	
31–40	2 (15.4%)	
51–60		2 (15.4%)
71–80		8 (61.5%)
81–90		3 (23.1%)
Gender		
Female	7 (53.8%)	10 (76.9%)
Male	6 (46.2%)	3 (23.1%)
Ethnicity		
African	1 (7.7%)	0
Asian	10 (76.9%)	6 (46.2%)
Hispanic/Latin American	1 (7.7%)	3 (23.1%)
Middle Eastern	1 (7.7%)	0
White/European descent	0	4 (30.8%)
Experience on any robots before		
Yes	7 (53.8%)	4 (30.8%)
No	6 (46.2%)	9 (69.2%)
Experience in tiny robots before		
Yes	1 (7.7%)	2 (15.4%)
No	12 (92.3%)	11 (84.6%)

Additional information specific to each group was also collected in the study. For the student group, the majority had an undergraduate educational background (84.6%), followed by a smaller proportion with graduate education (15.4%). In the older adult group, most participants were married (61.5%). Regarding self-rated health, most older adults rated their health as good (92.3%), with only one participant rating it as fair.

In terms of living arrangement, most older adults lived with family (46.2%), followed by those living with a partner (30.8%) and those living alone (23.1%). As for education level, most participants in the older adult group had a postgraduate education (53.8%).

Regarding conditions that may affect communication, eight participants reported no such conditions (61.5%), one participant reported experiencing mild cognitive impairment (MCI), short-term memory issues, hearing loss, and word retrieval difficulties (7.7%), and three participants preferred not to answer (23.1%). One participant indicated that the question was not applicable.

### 3.1 Emotional engagement and perceived value

Older adults emphasized the emotional and therapeutic value of the tiny social robots EMO and AIBI, describing moments of joy, reduced loneliness, and the uplifting effects of music and movement. They saw the robots as cheerful companions that brought warmth and affective presence into daily life. Nancy shared how her mother-in-law, who lives with dementia, lights up when interacting with EMO: “She likes to dance with EMO because it’s cheerful. Then she would dance with him.” For Nancy, EMO improves mood and encourages movement. Joan, another older adult, found it delightful when the robot encouraged light-hearted interruptions, saying, “It’s fun to see a robot dance and suddenly remind you to get up and move, have a dance party.” Others imagined how music could be used intentionally to enhance wellbeing. “Perhaps an older person could request a genre of music from their youth, smooth jazz, maybe something that creates a lovely ambience in the home and relieves anxiety,” said Frank, an older adult participant. “As far as I know, pet therapy works really well for people living with dementia. It helps ease depression and anxiety. I think these tiny robots could be quite similar because they are cute and comforting in the same way.” He envisioned the robot offering a personalized form of music therapy, stirring happy memories. The emotional engagement wasn’t just about what the robot did, but how it behaved. Margot, an older adult, affectionately referred to AIBI as “he” rather than “it,” remarking, “He’s upset,” when the robot displayed an angry expression. This subtle shift in language reflected a felt sense of companionship.

While older adults emphasized emotional warmth and companionship, university students viewed the robots more critically. Specifically, university students, while initially drawn to the cuteness and charm of the robots, were more critical of their emotional depth. This difference may relate to the students’ higher familiarity with technology, leading them to expect more complexity and spontaneity from the robots. Karina, a student, first commented on the robot’s appearance: “I think that the robot’s own appearance, it does have a cute look. I just wanted to say, I think the robot’s really good for the short term. It’s fun, it’s happy, but I feel like it’s quite empty for the long term.” She later expressed concerns about the interactions becoming monotonous: “It might become very repetitive. Like if I keep giving them tasks, they’ll just do exactly what I say. Personally, I would like it if there’s more randomness or spontaneity.” Michael, another student, echoed this concern, especially when thinking about long-term users: “It feels quite empty… for future use, like in long-term care, it might feel emotionally empty for older adults.” While students admired the expressive movements and responses, they worried the emotional engagement might fade without deeper interaction. These different reactions show how older adults appreciated even brief moments of cheer and connection in their daily routines, while students were more concerned with sustained novelty and variety. In summary, older adults valued the robots for the emotional comfort and sense of companionship they offered, whereas students focused on the limitations of emotional depth and long-term engagement.

### 3.2 Usefulness and functionality

Older adults and students both recognized the practical value of EMO and AIBI, though their perspectives differed in emphasis. Older adults focused more on relational and sensory access, emphasizing how the robots could support daily routines, offer reminders, and provide companionship in ways that felt intuitive and emotionally affirming. George, an older adult, suggested that the robot could remind him about medications, hydration, or exercise, saying, “If the robot reminded me to take my medicine or do some stretches, I think that would be helpful.” Nancy envisioned how Artificial Intelligence (AI) enabled robots could offer information in accessible ways: “My mother likes asking a lot of questions, but because of ChatGPT, they can now easily get the answers she wants like recipes for cooking or how to take medicine.” Some older participants also spoke about how the robot could reduce isolation: “The existence of a conversation from the robot to the person living alone is a great therapy and cutting the loneliness.” These comments highlight the desire for the robots to serve as both cognitive aids and socially supportive technologies. This focus on daily integration and emotional reassurance may reflect older adults’ lived experiences with routine, dependency, and the need for emotional continuity in later life.

University students shared similar insights but expressed deeper thought on the accessibility and convenience of the robots. Jane, a student, imagined how portability could help older adults maintain routines while on the go: “Smaller robots are very portable if they start their day with the same commands like ‘wake up’ or ‘take your medicine,’ it might be more convenient, even when they travel or move homes.” Several students, like Yuka, emphasized the benefits of visual movement for users with hearing loss: “The robot focuses on generating the response with movement, not sound… that’s useful for people like my grandpa who cannot hear well.” Students also imagined the robots recognizing family members, offering reminders about visits, or relaying wellness updates to caregivers. While their comments echoed some of the older adults’ expectations, students tended to interpret usefulness through a technological and design-oriented lens.

By contrast, students focused on technical and logistical constraints, often reflecting a caregiving or protective stance that emphasized what might go wrong if older adults were left alone with the robot. Precise wording of verbal commands was identified as a potential limitation to practical usability. Janna, a student, raised concerns about voice command failures: “Sometimes older adults with dementia… their verbal abilities can be impaired… and if the robot does not understand them, it could lead to frustration.” Another student, Jamie, worried about over-reliance as a potential risk: “If that thing fails, and you do not have a backup, it could be dangerous.” Karina suggested the robots might be more suitable for caregivers initially: “We should first give it to caregivers to help use this kind of robot… If the robot could imitate my grandpa’s voice and remind my grandma to eat, she’d love it.”

Overall, both groups viewed the robots as useful, but their priorities diverged: older adults valued emotional reassurance and intuitive usability, whereas students focused on system reliability, communication accuracy, and the potential for broader caregiving applications.

### 3.3 Risks and safety

While both older adults and students acknowledged the benefits of EMO and AIBI, they also identified a range of risks from different perspectives. Older adults focused on accessibility, sensory design, and usability. For example, Margot, an older adult, commented, “I would like them just to be one size bigger because I feel as though they might get lost in some of the clutter and to be just a bit more colourful.” Samira raised concerns about the robots’ sound quality: “The squeaky voice is hard to understand. Even in a quiet space, that was a very squeaky voice.” Leo, who has low vision, noted, “I would not be able to see the small screen, and if I cannot hear it clearly, I’m not sure what I’m supposed to do.” For Evelyn, tactile engagement mattered: “Especially if I was with someone in my family who had a bit of dementia, having textures and stuff is more sensory input.” Others noted the need for support and time to become comfortable with the robots. Clara shared, “It takes time to learn about how to make the robot work… and we need some support in that area for people who are living on their own.” These comments reflected a focus on maintaining comfort, clarity, and familiarity, which were shaped by their sensory experiences and their need for self-efficacy in daily life.

Students, on the other hand, highlighted technical breakdowns, miscommunication, and over-reliance, often from a caregiving or risk-avoidant stance. Jason and Alexis both worried about dependency: “What if someone really depends on the robot for their meds, and it shuts down? You do not want to rely on just one thing; if it fails, there needs to be a fallback.” William brought up maintenance concerns: “The battery dies fast, and older people might forget to charge it. Then it just sits there.” Physical risks also came up. Mei observed, “AIBI is so tiny if you drop it or leave it on the floor, someone could trip or step on it.” Jacob joked, “Honestly, I could see it ending up in someone’s laundry by mistake. It’s that small.”

Both groups mentioned language barriers and sensory challenges as serious limitations. Jane, a student, pointed out, “The robot only understands English. My grandparents speak Mandarin—so it just would not work.” Yuka echoed this, saying, “My grandfather is both visually and audibly impaired… the robot needs louder audio, or it’s useless for him.” Lauren noted that the rigid voice command system could be frustrating: “If you say something slightly off, it does not respond. I can imagine my grandmother giving up quickly.”

Finally, the social context of use was a concern. The robots were seen as best suited for individual interaction. Jason noted, “It might work well if you are just one-on-one, but in a group session like this, it did not work really well.” Yuka suggested, “Maybe if it could connect to a bigger screen, it would be easier for a group to follow along, especially during exercise or music time.”

In summary, older adults emphasized usability, sensory feedback, and the need for clear, supportive design. Students focused on reliability, maintenance, and the risks of failure or miscommunication. While their concerns differ, both groups highlighted the need for inclusive design that accommodates sensory impairments, linguistic diversity, and realistic use conditions, especially for older adults living alone or with cognitive challenges. These user-identified challenges highlight key usability barriers that need to be addressed in future co-design and technical refinement of social robots.

### 3.4 Intergenerational connection

Both older adults and university students described EMO and AIBI as unexpected bridges across generations, though the nature of this “bridge” differed between them. While older adults emphasized the emotional companionship and warmth the robots offered, students were more intrigued by how such technologies could mediate or enhance social connection. Participants from both groups observed that the robots’ facial expressions, movements, and playfulness created opportunities for interaction that felt genuine and emotionally resonant. Frank reflected, “What I like about robots is that they can work on our emotions and support that. If I feel down, they uplift me.” Student participant Alexis noted, “The robot would become happy with me. I think that would be positive and beneficial both ways.” This sense of mirrored emotion was echoed by Helen, an older adult, who remarked, “These facial expressions are really touching… they reach into people’s hearts.”

Participants contrasted this warmth with other technologies like Siri or ChatGPT on the phone, which were perceived as flat and impersonal. Hiroshi, an older adult, shared, “Many times, I need to talk with someone… but there’s no one. I turn on the TV or radio, but it’s not enough. I talk to Siri, but that’s just a voice. The robot is different; it has a face and expression.” Student Mei agreed: “Siri is cold. EMO and AIBI move, they react, and that matters more than words.” Older adults welcomed the idea of robots as a bridge for intergenerational engagement. They saw potential in shared play, co-learning, or parallel robot ownership that could enable social contact. Nancy further noted that robots might even attract social interaction from others: “It’s just like when you have a new cat or a new dog, people will come to you and ask questions, right? So it might bring people together.” She also imagined a broader digital connection, saying, “What if my friend had a robot and I had one? Maybe we could connect them somehow and talk, like a robot-to-robot bridge.” For her, the robot was not just a helper, but a partner in building connection. Susan noted, “Just having a conversation with the robot when you live alone makes a difference, it’s like having a little bit of company in the room.”

Students appreciated the role of robots in facilitating engagement but viewed them more as tools for enabling care or supplements to human interaction, not as a substitute for it. Student Michael envisioned using robots to prompt conversations or provide emotional reminders: “They could remind you of family stories, or memories you shared but we have to be careful. They should add to connection, not take its place.” Jason added, “You do not want people depending on robots for real social needs. That’s not what they are meant for.” Older adults imagined shared companionship, including the possibility of robots “talking” to each other across homes or helping initiate contact between friends or family members. Students were more cautious, highlighting that technology should augment, not replace, human relationships.

However, both groups acknowledged limitations to shared use, especially in group or communal settings. Tom suggested, “Maybe if it could connect to a bigger screen, it would be easier for a group to follow along, especially during exercise or music time.” Issues like low volume, squeaky voices, and tiny physical size also disrupted connection. George joked, “AIBI felt so tiny—my cat might steal it!” Jacob, a student, worried, “You might forget it in your pocket and find it in the washing machine.” Older participant Susan added, “Touch is important. But the robot’s not big enough to hug.” Despite these risks, both groups described how the robots created small, joyful moments that opened new ways of relating across age. The expressive, emotionally attuned design of EMO and AIBI invited curiosity, laughter, and shared attention, allowing older adults and students to see one another in new, more playful ways. While students focused more on design improvements, older adults leaned into the joy of companionship, imagining robots not only as tools, but as connectors of people and possibilities.

## 4 Discussion

This study aimed to explore the experiences and perceptions of older adults and university students interacting with social robots EMO and AIBI, particularly focusing on how these interactions fostered intergenerational connection and enhanced emotional and social engagement between different age groups. Our participants, including both the older adult group and the student group, expressed their views on the tiny robots, covering emotional aspects, practicality, risks and safety, as well as their potential impact on intergenerational relationships. Overall, after experiencing the tiny robots together, the participants showed a generally positive attitude towards them, which represents one of the key findings of this study. This result is consistent with previous studies showing that people generally hold positive attitudes toward social robots and are willing to engage with them (Naneva et al., 2020; Wullenkord and Eyssel, 2014). Our findings extend this evidence to tiny robots used in an intergenerational context.

Interestingly, in our study, most of the older adult participants were female. However, previous research has shown that, in the context of healthcare or caregiving robots, male older adults tend to have more positive attitudes than female older adults (Kuo et al., 2009; Stafford et al., 2014). Contrary to these findings, our study revealed that even female older adults exhibited high emotional acceptance of tiny social robots. This finding strengthens our confidence in applying this technology to a broader and more diverse population of older adults.

This study also found that while both older adults and students acknowledged the emotional support provided by tiny social robots, there were clear differences in the depth and criticality of their responses. Older adults focused more on the pleasurable experiences, emphasizing the interaction itself with the robots, while students demonstrated more critical and deeper reflections. This finding contrasts with previous research that suggests older adults may be more interested in functionality of the robots, such as helping with daily tasks, rather than focusing on their social capabilities (Søraa et al., 2023). Three explanations for this difference can be proposed: first, the difference may lie in how older participants described the robots in the focus group—unlike traditional social robots, tiny social robots are perceived as cuter, with more expressive faces, making it easier for older adults to empathize with them and see them as companions. Secondly, younger students are exposed to newer technology in their daily lives compared to older adults, which may be due to the “digital divide” (Volkom et al., 2014; Blažič and Blažič, 2020). As a result, older adults may approach emerging technologies like social robots with greater curiosity and are less likely to make rational comparisons with other technologies, as students tend to do. In essence, students might be more aware that varying levels of technological competence among older adults could influence their long-term interactions with the robot, based on the students’ observations of their grandparents, whereas the novelty of the robots was enough to capture the older adults’ interest during their first interaction. Finally, according to the Socioemotional Selectivity Theory (SST) (Carstensen et al., 1999), older adults are generally more inclined to view new things with a positive attitude. Research has shown that when robots make mistakes, regardless of the robot’s attitude towards admitting the error, the trust older adults place in the robot significantly decreases (Giorgi et al., 2022). This also supports the concerns expressed by students, as previous research similarly highlighted the critical importance of technology reliability for user trust and emphasized that this issue was worth paying attention to (Tian and Oviatt, 2021; Liu et al., 2023). Therefore, the perspectives of younger individuals provide a valuable complement to those of older adults, reminding them of limitations they may overlook when using robots. This highlights a key role in collaborative cooperation (Hung et al., 2024).

In terms of Usefulness and Functionality, older adults and university students showed different areas of focus. Older adults were more focused on the robots as memory aids with their built-in reminders and their functions as companions. Research indicates that when older adults face memory declines due to aging or subjective memory decline, they often adopt compensatory strategies to manage tasks that require memory in daily life (Carter et al., 2023). Currently, many technological supports focus on utilizing built-in smartphone functions or newly developed apps to provide targeted reminders for older adults (Andreassen et al., 2020; Hackett et al., 2022). However, the current study found that tiny robots can offer a different reminder experience compared to traditional technologies, incorporating emotional support and companionship functions. This may serve as an effective intervention, addressing both the cognitive and emotional needs of older adults with a simple solution.

In contrast to older adults, students tend to see robots primarily as tools, rather than companions, which highlights the role of emotional engagement in influencing perceptions of technological devices. Furthermore, students expressed concerns about over-reliance on robots, pointing to the potential risks of dependence on technology. These concerns align with findings from previous studies, which suggest that excessive reliance on technological interventions may weaken critical thinking and problem-solving skills (George et al., 2024). This is especially important in caregiving contexts. Therefore, while robots may offer tangible benefits in terms of reminders and support, careful consideration is needed to balance technological assistance with interpersonal interactions to ensure that robots enhance, rather than replace, meaningful social engagement (Hung et al., 2025).

Both older adults and university students in the current study acknowledged the unexpected role that EMO and AIBI played in fostering intergenerational connections. Both groups highlighted how the robots’ facial expressions and movements created opportunities for emotional connection, sparking emotional resonance between them. Older adults especially valued these warm interactions, seeing the robots not just as daily companions but also as social tools that help avoid loneliness. This perspective may be linked to the idea that different types of media elicit varying levels of emotional connection, with communication tools that convey multiple cues generally perceived as more effective than those that are purely text or audio, as suggested by the Media Richness Theory (Döring et al., 2024). Conversely, students recognized that the robots could promote conversation and emotional support, but they emphasized that these devices should act as tools to enhance human relationships rather than replace authentic social interaction. These findings suggest that, despite differing viewpoints, both groups recognized the interactive feature of the tiny robots as having potential to strengthen intergenerational connections. Previous studies have shown that incorporating social robots into home settings can serve as a catalyst for intergenerational communication (Chen et al., 2025), with older adults more willing to discuss family matters with robots they consider part of the family (Mo et al., 2017). This implies that tiny social robots may offer lasting companionships for older adults, helping to establish more sustainable and meaningful intergenerational relationships.

These findings have practical implications for dementia care. Tiny robots could be incorporated into daily activity programs or reminiscence therapy to enhance emotional engagement and social participation. Involving younger volunteers or students in robot-assisted activities may also promote intergenerational connection and user acceptance. Staff and caregivers could receive brief training on how to facilitate safe and meaningful interactions, ensuring technology complements rather than replaces human care.

### 4.1 Strengths and limitations

A key innovation of this study lies in its design for intergenerational interactions. Through focus group interviews, we gained deep insights into how older adults and university students perceived their intergenerational interactions while using the tiny social robots. This qualitative approach allowed us to capture participants’ emotional responses and individual differences, particularly in how the older adult group perceived the emotional support and practical functionality provided by the robots. Moreover, the study focused on exploring the potential of these robots to promote intergenerational interactions, which is crucial for the social engagement and emotional support of older adults.

One limitation of this study is the ethnicity imbalance in the sample, as participants were predominantly from Asian backgrounds, which may affect the generalizability of the findings. While researcher influence is an inherent consideration in qualitative research, this was addressed through a collaborative and reflexive analytic process under supervision, ensuring balanced interpretation. Additionally, there were health differences among the older adult participants, with some having MCI and hearing issues, which could have influenced their interaction experiences. Therefore, future research should expand the diversity of the sample and consider the impact of varying health conditions within the older adult population. Moreover, future studies should include participants from more diverse backgrounds, such as those living in rural areas, individuals with advanced dementia, and non-English-speaking older adults, to enhance the generalisability of findings. Finally, exploring the long-term usage and effects of interacting with the robots could provide valuable insights into their sustained impact on older adults’ social and emotional well-being. Future research should include longitudinal observation and larger, more diverse samples to examine long-term effects and generalizability. Co-design with caregivers and older adults could also help optimize robot features for practical use.

## 5 Conclusion

This study demonstrates that tiny social robots such as EMO and AIBI hold promise in enhancing emotional well-being and reducing social isolation among older adults, while also serving as catalysts for intergenerational connection. Although older adults valued their companionship and affective impact, younger participants highlighted concerns around technical reliability, usability, and over-reliance. These complementary perspectives underscore the need for co-design approaches that integrate the lived experiences of older adults with the critical insights of younger adults. Future research should expand to larger and more diverse populations and explore long-term use in home and community settings.

## Data Availability

The raw audio data are not publicly available to protect participants’ privacy, as the voices are personally identifiable and cannot be anonymized. Supporting anonymized transcripts are available from the corresponding author upon reasonable request.
