# Supplementary material for: Exploring intergenerational interactions with tiny social robots: a qualitative study
Source: Front Dement. 2025 Dec 4;4:1698659. doi: 10.3389/frdem.2025.1698659 (PMC12711846; doi:10.3389/frdem.2025.1698659)
Supplement: Supplementary file 1 [file Supplementary_file_1.docx]

COREQ (Consolidated Criteria for Reporting Qualitative Research): 32-item Checklist

Adapted from : Tong, A., Sainsbury, P., & Craig, J. (2007). Consolidated criteria for reporting qualitative research (COREQ): a 32-item checklist for interviews and focus groups. *International journal for quality in health care*, 19(6), 349-357.

| Domain and Item | Guide Questions / Description | Section where Addressed |
| --- | --- | --- |
| Domain 1: Research team and reflexivity |  |  |
| 1. Interviewer/facilitator | Which author/s conducted the interview or focus group? | Section 2.5, Data Collection |
| 2. Credentials | What were the researcher’s credentials? (e.g., PhD, MD) | Section 2.5, Data Collection |
| 3. Occupation | What was their occupation at the time of the study? | Section 2.5, Data Collection |
| 4. Gender | Was the researcher male or female? | Section 2.5, Data Collection |
| 5. Experience and training | What experience or training did the researcher have? | Section 2.5, Data Collection |
| 6. Relationship established | Was a relationship established prior to study commencement? | Section 2.7 Patient and Public Involvement |
| 7. Participant knowledge of the interviewer | What did the participants know about the researcher? (e.g., personal goals, reasons for doing the research) | Section 2.8 Ethical Considerations |
| 8. Interviewer characteristics | What characteristics were reported about the interviewer/facilitator (e.g., bias, assumptions, reasons, interests in the research topic)? | Section 2.5 Data Collection |
| Domain 2: Study design |  |  |
| 9. Methodological orientation and theory | What methodological orientation was stated to underpin the study? (e.g., grounded theory, discourse analysis, ethnography, phenomenology, content analysis) | Section 2.1 Design, paragraph 1 |
| 10. Sampling | How were participants selected? (e.g., purposive, convenience, snowball) | Section 2.3 Sampling and Recruitment, paragraph 1 |
| 11. Method of approach | How were participants approached? (e.g., face-to-face, telephone, mail, email) | Section 2.3 Sampling and Recruitment, paragraph 1 |
| 12. Sample size | How many participants were in the study? | Section 2.3 Sampling and Recruitment, paragraph 1 |
| 13. Non-participation | How many people refused to participate or dropped out? Reasons? | Section 2.3 Sampling and Recruitment, paragraph 1 |
| 14. Setting of data collection | Where was the data collected? (e.g., home, clinic, workplace) | Section 2.2 Study Settings and the intervention |
| 15. Presence of non-participants | Was anyone else present besides the participants and researchers? | Section 2.5 Data collection |
| 16. Description of sample | What are the important characteristics of the sample (e.g., demographic data, date) | Section 3 Results, paragraph 1: Table 2 |
| 17. Interview guide | Were questions, prompts, guides provided by the authors? Was it pilot tested? | Section 2.5 Data Collection |
| 18. Repeat interviews | Were repeat interviews carried out? If yes, how many? | Not applicable (no repeat interviews were conducted) |
| 19. Audio/visual recording | Did the research use audio or visual recording to collect the data? | Section 2.5 Data Collection |
| 20. Field notes | Were field notes made during and/or after the interview or focus group? | Section 2.5 Data Collection |
| 21. Duration | What was the duration of the interviews or focus groups? | Section 2.5 Data Collection |
| 22. Data saturation | Was data saturation discussed? | Not applicable (one-time focus group study) |
| 23. Transcripts returned | Were transcripts returned to participants for comment and/or correction? | Not applicable (Section 2.7 Patient and public involvement) |
| Domain 3: Analysis and findings |  |  |
| 24. Number of data coders | How many data coders coded the data? | Section 2.6 Data Analysis and Theoretical Framework |
| 25. Description of the coding tree | Did authors provide a description of the coding tree? | Section 2.6 Data Analysis and Theoretical Framework: Table 1 |
| 26. Derivation of themes | Were themes identified in advance or derived from the data? | Section 2.6 Data Analysis and Theoretical Framework |
| 27. Software | What software, if applicable, was used to manage the data? | Section 2.6 Data Analysis and Theoretical Framework |
| 28. Participant checking | Did participants provide feedback on the findings? | Not applicable (Section 2.7 Patient and public involvement) |
| 29. Quotations presented | Were participant quotations presented to illustrate the themes/findings? Was each quotation identified? | Section 3 Results |
| 30. Data and findings consistent | Was there consistency between the data presented and the findings? | Section 3 Results and Section 4 Discussion |
| 31. Clarity of major themes | Were major themes clearly presented in the findings? | Section 3 Results |
| 32. Clarity of minor themes | Is there a description of diverse cases or discussion of minor themes? | Section 3 Results |
